# Supplementary material for: Bayesian refinement of protein structures and ensembles against SAXS data using molecular dynamics
Source: PLoS Comput Biol. 2017 Oct 18;13(10):e1005800. doi: 10.1371/journal.pcbi.1005800 (PMC5662244; doi:10.1371/journal.pcbi.1005800)
Supplement: S1 Archive — (BZ2) [file pcbi.1005800.s011.tar.bz2 › gromacs_4.62_bayesian_saxs_refi/share/html/online/pdb2gmx.html]

pdb2gmx


| pdb2gmx Main Table of Contents |

**VERSION 4.6  
Sat 19 Jan 2013**

---

### Description

This program reads a .pdb (or .gro) file, reads
some database files, adds hydrogens to the molecules and generates
coordinates in GROMACS (GROMOS), or optionally .pdb, format
and a topology in GROMACS format.
These files can subsequently be processed to generate a run input file.

pdb2gmx will search for force fields by looking for
a forcefield.itp file in subdirectories <forcefield>.ff
of the current working directory and of the GROMACS library directory
as inferred from the path of the binary or the GMXLIB environment
variable.
By default the forcefield selection is interactive,
but you can use the -ff option to specify one of the short names
in the list on the command line instead. In that case pdb2gmx just looks
for the corresponding <forcefield>.ff directory.

After choosing a force field, all files will be read only from
the corresponding force field directory.
If you want to modify or add a residue types, you can copy the force
field directory from the GROMACS library directory to your current
working directory. If you want to add new protein residue types,
you will need to modify residuetypes.dat in the library directory
or copy the whole library directory to a local directory and set
the environment variable GMXLIB to the name of that directory.
Check Chapter 5 of the manual for more information about file formats.

Note that a .pdb file is nothing more than a file format, and it
need not necessarily contain a protein structure. Every kind of
molecule for which there is support in the database can be converted.
If there is no support in the database, you can add it yourself.

The program has limited intelligence, it reads a number of database
files, that allow it to make special bonds (Cys-Cys, Heme-His, etc.),
if necessary this can be done manually. The program can prompt the
user to select which kind of LYS, ASP, GLU, CYS or HIS residue is
desired. For Lys the choice is between neutral (two protons on NZ) or
protonated (three protons, default), for Asp and Glu unprotonated
(default) or protonated, for His the proton can be either on ND1,
on NE2 or on both. By default these selections are done automatically.
For His, this is based on an optimal hydrogen bonding
conformation. Hydrogen bonds are defined based on a simple geometric
criterion, specified by the maximum hydrogen-donor-acceptor angle
and donor-acceptor distance, which are set by -angle and
-dist respectively.

The protonation state of N- and C-termini can be chosen interactively
with the -ter flag. Default termini are ionized (NH3+ and COO-),
respectively. Some force fields support zwitterionic forms for chains of
one residue, but for polypeptides these options should NOT be selected.
The AMBER force fields have unique forms for the terminal residues,
and these are incompatible with the -ter mechanism. You need
to prefix your N- or C-terminal residue names with "N" or "C"
respectively to use these forms, making sure you preserve the format
of the coordinate file. Alternatively, use named terminating residues
(e.g. ACE, NME).

The separation of chains is not entirely trivial since the markup
in user-generated PDB files frequently varies and sometimes it
is desirable to merge entries across a TER record, for instance
if you want a disulfide bridge or distance restraints between
two protein chains or if you have a HEME group bound to a protein.
In such cases multiple chains should be contained in a single
moleculetype definition.
To handle this, pdb2gmx uses two separate options.
First, -chainsep allows you to choose when a new chemical chain should
start, and termini added when applicable. This can be done based on the
existence of TER records, when the chain id changes, or combinations of either
or both of these. You can also do the selection fully interactively.
In addition, there is a -merge option that controls how multiple chains
are merged into one moleculetype, after adding all the chemical termini (or not).
This can be turned off (no merging), all non-water chains can be merged into a
single molecule, or the selection can be done interactively.

pdb2gmx will also check the occupancy field of the .pdb file.
If any of the occupancies are not one, indicating that the atom is
not resolved well in the structure, a warning message is issued.
When a .pdb file does not originate from an X-ray structure determination
all occupancy fields may be zero. Either way, it is up to the user
to verify the correctness of the input data (read the article!).

During processing the atoms will be reordered according to GROMACS
conventions. With -n an index file can be generated that
contains one group reordered in the same way. This allows you to
convert a GROMOS trajectory and coordinate file to GROMOS. There is
one limitation: reordering is done after the hydrogens are stripped
from the input and before new hydrogens are added. This means that
you should not use -ignh.

The .gro and .g96 file formats do not support chain
identifiers. Therefore it is useful to enter a .pdb file name at
the -o option when you want to convert a multi-chain .pdb file.

The option -vsite removes hydrogen and fast improper dihedral
motions. Angular and out-of-plane motions can be removed by changing
hydrogens into virtual sites and fixing angles, which fixes their
position relative to neighboring atoms. Additionally, all atoms in the
aromatic rings of the standard amino acids (i.e. PHE, TRP, TYR and HIS)
can be converted into virtual sites, eliminating the fast improper dihedral
fluctuations in these rings. **Note** that in this case all other hydrogen
atoms are also converted to virtual sites. The mass of all atoms that are
converted into virtual sites, is added to the heavy atoms.

Also slowing down of dihedral motion can be done with -heavyh
done by increasing the hydrogen-mass by a factor of 4. This is also
done for water hydrogens to slow down the rotational motion of water.
The increase in mass of the hydrogens is subtracted from the bonded
(heavy) atom so that the total mass of the system remains the same.

### Files

| option | filename | type | description |
| --- | --- | --- | --- |
| **-f** | eiwit.pdb | Input | Structure file: gro g96 pdb tpr etc. |
| **-o** | conf.gro | Output | Structure file: gro g96 pdb etc. |
| **-p** | topol.top | Output | Topology file |
| **-i** | posre.itp | Output | Include file for topology |
| **-n** | clean.ndx | Output, Opt. | Index file |
| **-q** | clean.pdb | Output, Opt. | Structure file: gro g96 pdb etc. |

### Other options

| option | type | default | description |
| --- | --- | --- | --- |
| **-[no]h** | bool | no | Print help info and quit || **-[no]version** | bool | no | Print version info and quit || **-nice** | int | 0 | Set the nicelevel || **-chainsep** | enum | id\_or\_ter | Condition in PDB files when a new chain should be started (adding termini): id\_or\_ter, id\_and\_ter, ter, id or interactive || **-merge** | enum | no | Merge multiple chains into a single [moleculetype]: no, all or interactive || **-ff** | string | select | Force field, interactive by default. Use -h for information. || **-water** | enum | select | Water model to use: select, none, spc, spce, tip3p, tip4p or tip5p || **-[no]inter** | bool | no | Set the next 8 options to interactive || **-[no]ss** | bool | no | Interactive SS bridge selection || **-[no]ter** | bool | no | Interactive termini selection, instead of charged (default) || **-[no]lys** | bool | no | Interactive lysine selection, instead of charged || **-[no]arg** | bool | no | Interactive arginine selection, instead of charged || **-[no]asp** | bool | no | Interactive aspartic acid selection, instead of charged || **-[no]glu** | bool | no | Interactive glutamic acid selection, instead of charged || **-[no]gln** | bool | no | Interactive glutamine selection, instead of neutral || **-[no]his** | bool | no | Interactive histidine selection, instead of checking H-bonds || **-angle** | real | 135 | Minimum hydrogen-donor-acceptor angle for a H-bond (degrees) || **-dist** | real | 0.3 | Maximum donor-acceptor distance for a H-bond (nm) || **-[no]una** | bool | no | Select aromatic rings with united CH atoms on phenylalanine, tryptophane and tyrosine || **-[no]ignh** | bool | no | Ignore hydrogen atoms that are in the coordinate file || **-[no]missing** | bool | no | Continue when atoms are missing, dangerous || **-[no]v** | bool | no | Be slightly more verbose in messages || **-posrefc** | real | 1000 | Force constant for position restraints || **-vsite** | enum | none | Convert atoms to virtual sites: none, hydrogens or aromatics || **-[no]heavyh** | bool | no | Make hydrogen atoms heavy || **-[no]deuterate** | bool | no | Change the mass of hydrogens to 2 amu || **-[no]chargegrp** | bool | yes | Use charge groups in the .rtp file || **-[no]cmap** | bool | yes | Use cmap torsions (if enabled in the .rtp file) || **-[no]renum** | bool | no | Renumber the residues consecutively in the output || **-[no]rtpres** | bool | no | Use .rtp entry names as residue names |

---

http://www.gromacs.org  
gromacs@gromacs.org
